# Association between serum ferritin level and diastolic cardiac function in patients with major β-thalassemia

**Published:** 2015-04-20

**Authors:** A Eghbali, H Taherahmadi, B Bagheri, S Nikanjam, L Ebrahimi

**Affiliations:** 1 Pediatric Hematologist & Oncologist, Department of Pediatrics, Arak University of Medical Sciences, Arak, Iran.; 2 Department of Pediatrics, Arak University of Medical Sciences, Arak, Iran.; 3 Cancer Research Center and Department of Pharmacology, Semnan University of Medical sciences, Semnan, Iran.; 4 Student of Medicine, Arak University of Medical Sciences, Arak, Iran.; 5 Blood transfusion research center, High Institute for Research and Education in Transfusion Medicine, Tehran, Iran.

**Keywords:** Diastolic Dysfunction, Echocardiography, Ejection Fraction, Ferritin, Thalassemia

## Abstract

**Background:**

Prevention of myocardial siderosis is a key step to reduce rate of mortality in thalassemic patients. Our objective was to study association between echocardiography parameters and serum ferritin level in patients with major thalassemia.

**Materials and Methods:**

Sixty-six patients with major thalassemia were studied in Amir Kabir hospital, Arak, Iran. Serum ferritin levels were measured during 3 months in patients with no symptoms of infection. It was measured by enzyme-linked immunosorbent assay (ELISA). Ejection Fraction (EF), Fractional Shortening (FS) and Early/Late ratio (E/A) were studied by echocardiography.

**Results:**

Fifty two percent were female and 48% were male. Mean age was 16 ± 9 years and the age range was3-26years. Mean serum ferritin level was1912 ± 1748 ng/dl and its range was from 303 to 8333 ng/dl. There were significant correlations between serum ferritin level and EF(r=0.3 and P=0.05) and also between serum ferritin level and FS.

**Conclusion:**

Due to significant association between serum ferritin level and echo parameters, it is beneficial that all patients with major thalassemia undergo echocardiography to gain better understating about cardiac function.

## Introduction

Heart failure secondary to iron over load is the main cause of death in patients with major thalassemia. Most of patients suffer from myocardial fibrosis and cardiac dysfunction due to overload of iron in cardiac tissues (1,2). Iron chelating may reduce cardiac iron overload and reduce rate of mortality in such patients. Heart injuries in iron overloud cases include dilatation of the atria and ventricles, arrhythmia, valvular dysfunction, pericarditis, thickening of the muscles and finally heart failure (2,3). Although frequent blood transfusion is routinely done, patients suffer from chest pain, tachycardia, exhausting and in some cases sudden death. In order to have an accurate 

iron chelating, it is necessary to measure the amount of patient’s iron. The most accurate method to measure iron level is liver biopsy; however, it is invasive and unable to provide an accurate measurement of heart iron level (4). During recent years, non-invasive methods have gained prominence. T2* MRI can measure iron levels in the heart and liver. But this methods is not readily available everywhere and is not applicable to all cases and is useless for evaluation of cardiac function (5). A great deal of inertest has centered on cardiac function that can be evaluated by echocardiography. Some studies have proved that serum ferritin cannot show total iron level especially in the heart (6,7). On the other hand, some researchers have agreed that serum ferritin is associated with cardiac iron level (8,9). In addition, they suggest that measurement of cardiac enzymes; Electrocardiography and echocardiography are useful methods for better evaluation of heart function. Other investigations showed that echocardiography parameters like Early/Late (E/A), Ejection Fraction (EF) and Fractional Shortening (FS) were associated with serum ferritin. 

Because current findings are not conclusive, the present study was designed to find a possible association between serum level of ferritin and echo findings in patients with β–thalassemia. 

## Materials and Methods

**Study subjects**

This cross sectional and descriptive study was done in Amir Kabir hospital, Arak, Iran. Sixty-six accessible children with major β–thalassemia who were above 2 year-old were included in the study from April 2014 to December 2014. Data were gathered through interview, clinical examination, detection of serum ferritin and echocardiography. The inclusion criterion was patients who suffer from major β–thalassemia for more than 2 years and exclusion criteria were children younger than 2 year-old and patients with hepatic complications. Clinical characteristics of the patients, age of blood transfusion initiation, age of chelator therapy initiation, and interval between blood transfusions, serum ferritin level and E/A, FS and EF were recorded in questionnaire.Serum ferritin levels were measured during 3 months in patients with no symptoms of infection. All patients received deferoxamine (25-60 mg/kg) or deferasirox (20-40 mg/kg). The exact dose of iron chelators was determined according to ferritin levels of patients. Informed consent letter was taken from parents of the patients and local ethical committee approved the study.

**Measurement of ferritin serum levels**

 A sandwich enzyme-linked immunosorbent assay (ELISA) was performed (Ray Bio, US). In short, 100µl serum was added to microtiter plates. The incubation time was 2.5 hours at room temperature. After that, 100µl prepared biothin antibody was added to each well and incubated for 1 hour at room temperature. Then, 100µl streptavidin solution was added and incubated for 45 minutes at room temperature. The intensity of the color was measured at 450 nm by Stat Fax 2600 (Awareness Technology, US) plate reader. Stat Fax 2100 (Awareness Technology, US) was used for washing steps. Next, mean serum ferritin was recorded during last 3 months. serum ferritin was measured in patients who showed no evidence of bacterial or viral infection.

**Echocardiography**

EF (Ejection Fraction): is a measurement of blood amount that left ventricle pumps out with each contraction (Normal range: 54-57%). FS (Fractional Shortening): is the fraction of any diastolic dimension that is lost in systole and calculated by this formula: (LVEDD - LVESD / LVEDD) x 100 (Normal range: 28-40%). E/A: (Early/Late) is a marker of the function of the left ventricle of the heart; it is determined on echocardiography with normal 1/9. 

**Statistical analysis**

Data are reported as mean ± SD. T test was used to measure difference between two independent variables. Categorical variables were compared using one-way analysis of variance. The correlations between parameters were determined using Pearson test. Probability value < 0.05 was considered to denote significant differences. All analyses were performed by SPSS 16.

## Results

Clinical characteristics

Table I shows clinical characteristics of the study subjects. Sixty six patients with major thalassemia were studied. Thirty four (52%) were female and 32 (48%) were male. Range of age was 3 to 46 years old with mean of 19 ± 9 yr. Patients were divided into 4 age groups (yr): <5, 5-10, 10-15 and > 15. Blood transfusion intervals were from 18 to 60 days with mean of 19 ± 7 days.

**Serum levels of ferritin**

Mean level of serum ferritin were calculated within 3 months. It was1912 ± 1748 ng/dl and its range was from 303 to 8333 ng/dl. According to 4 age groups (<5, 5-10, 10-15 and > 15yr), mean level of serum ferritin were: 425 ± 122 ng/dl, 785 ± 241 ng/dl, 1810 ± 1354 ng/dl and 3400 ± 2947 ng/dl, respectively. Older patients had higher ferritn levels.

 Ferritn levels were lower than 2500 mg/dl in 50 patients, between 2500 to 5000 ng/dl in 13 patients and above 5000 ng/dl in 3 patients.

**Echo findings**

**Figure 1:**
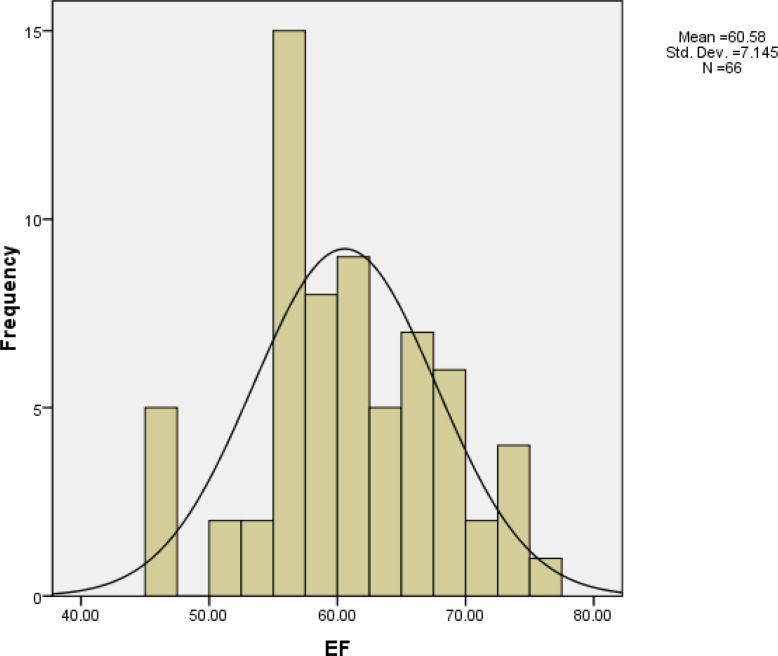
Distribution of EF in 66 patients

**Figure 2:**
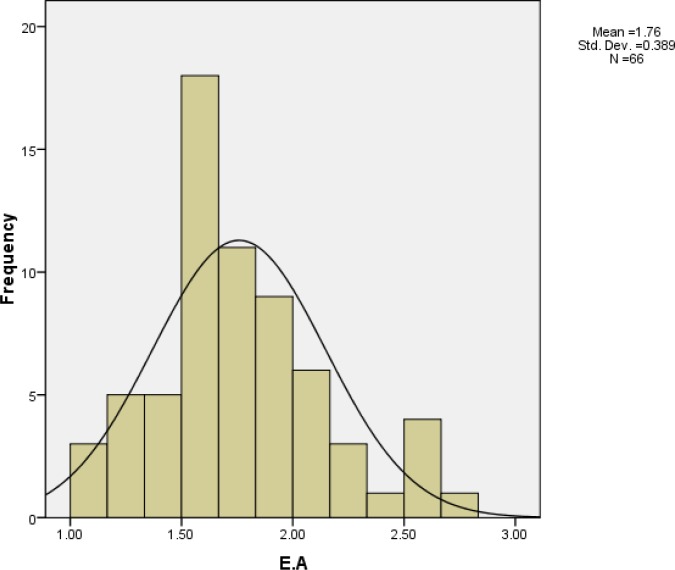
Distribution of EA in 66 patients

Range of EF was 45% to 75% and its mean was 60 ± 7 %. EF distribution is shown in Figure 1. Range of EA was 1% to 2% and its mean was 1.8 ± 0.4%. EA distribution is shown in Figure 2.

**Pearson correlations**

**Figure 3:**
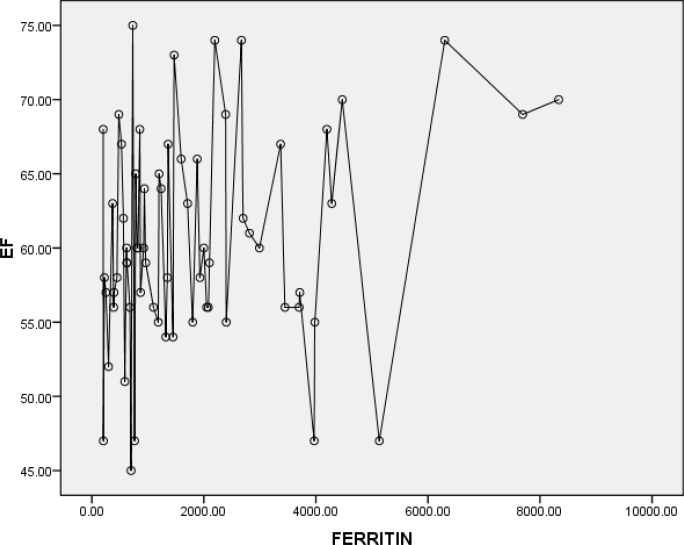
EF and Ferritin correlation* (r*=0.3 and *P*=0.05)

**Figure 4:**
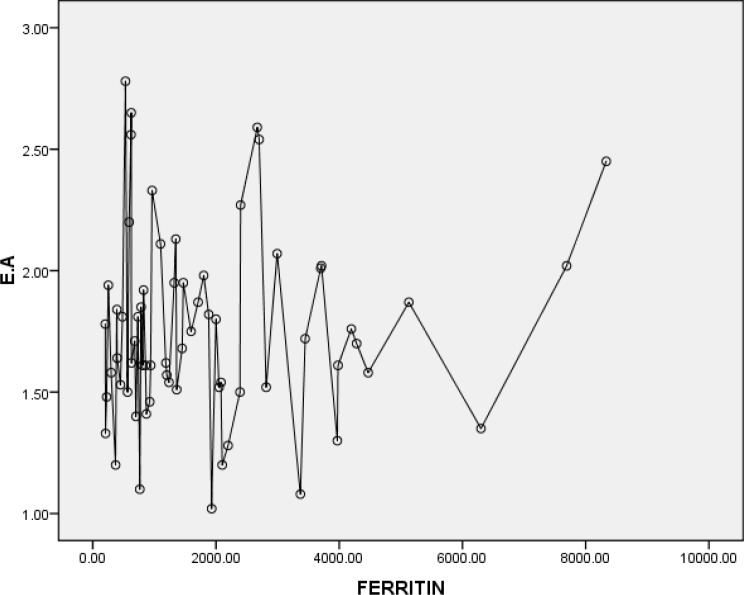
EA and Ferritin correlation (*r*=0.3 and *P*=0.05).

 A significant correlation was noted between serum ferritin level and EF(r=0.3 and P=0.05) and also between serum ferritin level and FS (r=0.2 and P=0.05) (Figure 3 and 4, respectively). Moreover, significant correlations were seen between sex with EF and FS, respectively (r= 0.2 and P=0.05).

**Table I:** Clinical characteristics of 66 patients

**Characteristic**	**Value**
**Age (yr)**	19 ± 9
**Male**	32
**Female**	34
**Age of Blood transfusion (yr)**	2 ± 1
**Age of chelator therapy (yr)**	4 ± 3

Data are presented as Mean ± SD or percentage.

## Discussion

β-thalassemic patients are susceptible to iron overload because of increase in absorption of iron and frequent blood transfusion (4). In recent years, many methods have been taken into consideration for the exact measurement of iron level. One of the indirect method is measurement of serum ferritin which has no value in inflammatory disorders or hepatic involvement (10). Echocardiography is useful to study anatomical changes of the heart. However, functional changes are usually observed in advanced stages of cardiomyopathy (11). In the present study, we used echocardiography and also measured serum levels of ferritin in 66 patients with β-thalassemia. Mean ferritin was 1912 ± 1748 ng/dl. Mean EF was 60 ± 7.1 % and mean FS was 32 ± 5.1 %. Moreover, mean E/A was 1.8 ± 0.4. We also found weak but significant association between ferritin level and echo parameters. A study showed that ferritin was associated with echo findings like E/A, EF and FS (12). Another study proved that patients who had high levels of ferritin, presented with higher LVDdt and lower EF and FS (13). Similar investigation proved that all patients, who were on deferoxamine, showed improvement in LVEF (14). In line with these studies, our findings showed that increase in ferritin level was correlated with increase of EF and FS. Ferritin subdivided into less than 2500, between 2500-5000 and more than 5000 did not reach significant difference. A study demonstrated that patients with less than 2500 ferritin had normal systolic and diastolic function, yet patients who had high levels of ferritin showed decrease in Declaration Time (DT); increase in E/Em (early ventricular filling velocity/early diastole myocardial velocity) and Mitral A peak (15). Furthermore, another cohort study showed no correlation between iron levels of the liver and LVEF

(16). In line with these findings, Tanner and colleagues revealed no significant correlation between LVEF and iron levels of the liver (17,18). A similar study on Iranian children showed no significant correlation between echo findings and ferrtin levels (19). It is noteworthy that diastolic dysfunctions occur at early stages of siderrosis (20). Therefore, in thalassemic patients (A) peak velocity in late diastole increases and E/A (Early & Late peak of flow velocity) decreases. We understood that ferritin had no correlation with E/A and EFm, yet ferritin had significant correlation with FS (R=0.563).

## Conclusion

In concert with other investigations, our study has provided evidence for accurate evaluation of iron level in thalassemic patients. It should be noted that due to factors affecting the serum level of ferritin, it is concluded that measurement of serum ferritin is not of great value in evaluation of cardiac function. Importantly, we suggest thalassemic patients undergo echocardiography to have adequate assessment of cardiac function. 
